# Differences in perceived chromatic aberration between emmetropic and myopic eyes using adaptive optics

**DOI:** 10.3389/fmed.2025.1504560

**Published:** 2025-08-04

**Authors:** Victor Rodriguez-Lopez, Paulina Dotor-Goytia, Elena Moreno, Maria Vinas-Pena

**Affiliations:** Institute of Optics, Spanish National Research Council (IO-CSIC), Madrid, Spain

**Keywords:** myopia, chromatic defocus, longitudinal chromatic aberration, blur perception, refractive error, adaptive optics, monochromatic high-order aberrations

## Abstract

**Introduction:**

The study of polychromatic visual perception is challenging due to the number of entangled factors involved in the process, from the cues within visual information from the outside world, to the ocular optics, the retinal properties, and neural adaptation processes in the brain.

**Methods:**

In this study, we used an adaptive optics (AO)- based polychromatic visual simulator to investigate the perception of combined optical cues and its dependence on refractive error. Subjective best focus was obtained as the average of 3 repeated measurements for (1) polychromatic and five monochromatic wavelengths in the visible (450–670 nm); (2) three different visual stimuli (conventional binary sunburst, natural outdoor image, natural indoor image); and (3) under natural aberrations (no-AO) and corrected aberrations (AO) conditions. Repeatability was determined as the standard deviation across repetitions. Chromatic difference of focus (CDF) was calculated for Green-Blue (G-Blue, 550–470 nm) and Green-Red (G-Red, 550–700 nm). Longitudinal chromatic aberration (LCA) was estimated using a polynomial regression fit of the best subjective focus curves as a function of the wavelength. Nine young adults (28 ± 6 years) with different refractive profiles (6 myopic and 3 emmetropic) participated in this study.

**Results:**

CDF showed different trends in the G-Red and the G-Blue regions, especially for the binary stimulus and after AO-correction of aberrations. However, in the myopic group, CDF was similar in absolute value for G-Blue and G-Red (0.61 ± 0.34 and 0.73 ± 0.58, respectively, *p* > 0.05 Mann-Whitney U test), whereas, in the emmetropic group, the chromatic difference was greater for G-Blue than for G-Red (0.58 ± 0.32 D and 0.22 ± 0.38 D, respectively, *p* < 0.05 Mann-Whitney U test). There was no effect of correcting natural aberrations. LCA does not vary with refractive error.

**Discussion:**

Overall, the results of this study suggest that the refractive profile may influence how visual information with specific chromatic properties is perceived and processed, potentially shaping visual mechanisms involved in chromatic defocus perception.

## 1 Introduction

The study of polychromatic visual information processing is challenging. First, visual inputs from the outside world are rarely well-defined homogeneous patches of light or dark, but are typically a mixture of polychromatic large-and small-scale structures (1–3) that interact with the dynamics of visual function (i.e., accommodation, binocular vision, eye movements, and adaptation, among others) (4–8). Furthermore, ocular optics is far from being a diffraction-limited system, and retinal image quality is degraded by the effects of monochromatic and chromatic aberrations, and their interactions (9–11). Finally, prolonged exposure to a degraded stimulus (i.e., a blurred retinal image) alters visual perception (12, 13) by shifting blur discrimination thresholds, more prominently in myopes than in emmetropes (14, 15).

Visual information processing, which is modulated by ocular dynamics, is essential for defocus detection (16, 17) and thus for vision. Accommodation, monochromatic high-order aberrations (HOAs), peripheral defocus, astigmatism, and chromatic aberrations are thought to modulate the sign and magnitude of defocus on the retina, and may also alter the temporal and spatial integration of defocus signals across the retina (18). Optical defocus leads to a proportional degradation of contrast at the edges of the images, a potential cue for the retina that would use edge contrast to determine the focal plane, and color contrast to identify the sign of defocus (3). However, retinal blur has been identified as the primary even-error stimulus for accommodation (19–21), the accommodative response becomes more precise when aberrations are corrected, and the presence of higher amounts of HOAs produces an increase in accommodative lag (22), similar to the increased accommodative lag found in myopes (23).

Ocular aberrations also play an important role in visual perception, and their correction could either improve retinal image quality and visual performance (24, 25) or reduce it due to adaptation. One study (26) reported that the presence of HOAs results in different point spread functions for hyperopic and myopic defocus, suggesting that these differences may be used by the visual system to determine the correct direction of focus shift. However, the relationship between aberrations and refractive error is inconclusive. Some studies have suggested a slightly higher amount of monochromatic aberrations in myopic eyes (27–29), while others have found no correlation between aberrations and refractive error (30–34) and others have found higher levels of HOAs in hyperopes (35). It seems likely that increased axial growth is accompanied by geometrical changes in the ocular components, resulting in changes in the aberration pattern and magnitude (36). In any case, if increased high-order aberrations occur in myopes, they seem to be more pronounced in high myopia (37), and related to structural changes.

In addition, chromatic cues may aid in detecting the sign of defocus. Broadband light produces color fringes on the retinal image, which may provide a signed chromatic signal of whether the defocus is hyperopic or myopic (3), as shown in a variety of animal models (18). Chromatic dispersion causes short wavelengths to focus in front of long wavelengths, creating a chromatic focus difference between them, known as longitudinal or axial chromatic aberration (LCA) (38). LCA causes long wavelengths to be focused on a more hyperopic plane than shorter wavelengths, so that the total refraction of the eye varies inversely with wavelength. Ocular LCA shows low intersubject variability, with subjective LCA (≈ 2D in the visible range) being significantly higher (by 0.50 D in the 488–700 nm range) than objective LCA (measured using reflectometric methods), likely due to differences in the retinal reflection planes and the retinal image focal plane (39, 40). Polychromatic optical quality in the phakic eye depends on the delicate balance between monochromatic and chromatic aberrations (41–43), which may be an important factor in myopic eyes because the human eye can use chromatic defocus as a directional cue for accommodation to both moving and stationary objects (44, 45). However, some individuals are able to accommodate in the absence of chromatic aberration, suggesting the existence of other achromatic cues that drive accommodation (46). Recently, Swiatczak and Schaeffel suggested that the human retina uses this difference in focus in the blue and the red to determine the sign of defocus, and hypothesized that the myopic retina has lost the ability to respond to LCA (47). Strikingly, the amount of both subjective and objective LCA was independent of the presence of HOAs (39), at least in a young emmetropic sample.

Adaptive optics (AO) based visual simulators operating at multiple wavelengths, with complementary AO elements for blur manipulation, allow to simulate vision under very different conditions, using a variety of psychophysical paradigms (i.e., method of limits, constant stimuli, adaptive staircase methods, among others), and artificial and naturalistic stimuli (Gabor patches, gratings, letters, natural images). AO allows to explore the limits of spatial vision imposed by the ocular optics (24) and to bypass them to study the neural adaptation processes in the brain, as well as to test the visual response to different optical cues in combination with relevant ocular properties (i.e., aberrations, accommodation, neural adaptation). Recently, a filter-based Badal LCA compensator incorporated into an AO scanning laser ophthalmoscope (AOSLO) allowed the independent and simultaneous control of focus at different wavelengths, so it can be tuned to compensate for the LCA of each individual eye (48), paving the way for more effective ways to modulate LCA, and understand its impact. The aim of this study is to investigate differences in the visual perception of stimuli with different spectral and spatial content in monochromatic and chromatic conditions, while controlling the subject’s monochromatic high-order aberrations using an AO-based polychromatic visual simulator. In particular, we investigate differences in the perceived best focus of subjects with different refractive errors in young adults.

## 2 Methods

Through-focus (TF) optical and visual quality with different stimuli (binary black and white, grayscale natural images) using five monochromatic and one polychromatic conditions was tested in a polychromatic (AO) based simulator. The subjective best focus for the same conditions was measured in 9 subjects, with different refractive errors, while controlling their ocular aberrations.

### 2.1 Stimuli: binary and natural images

**Figure 1:**
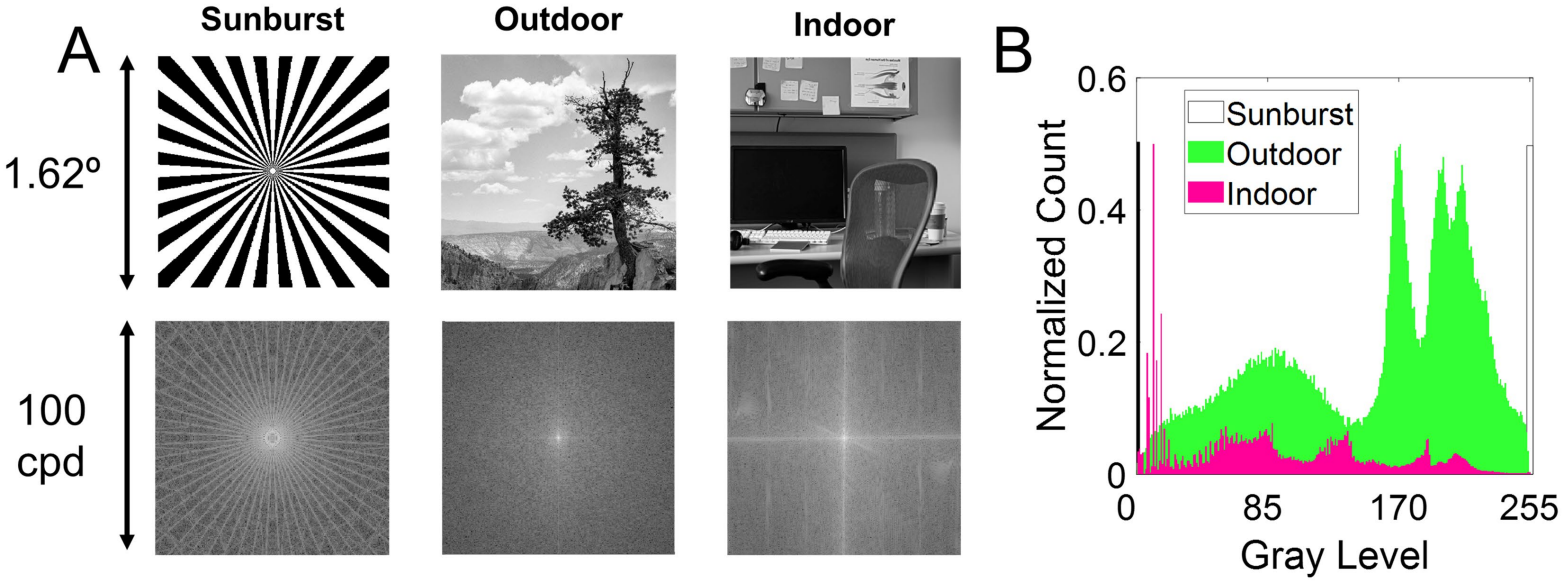
Visual stimuli. **(A)** Top row, stimuli used in the study. Bottom row, the frequency spectrum calculated as the magnitude of the Fourier transform. The stimuli are (1) Sunburst, a binary mask; (2) Outdoor, a natural grayscale image depicting an outdoor scene of a distant tree; and (3) Indoor, a natural grayscale image depicting an indoor scene of an office. **(B)** Histogram of the three images.

Three different stimuli were used to evaluate the influence of visual information on the perception of the best focus in monochromatic and polychromatic conditions. Figure 1A shows the three stimuli used in the study (top row) and their corresponding frequency spectrum calculated as the magnitude of the Fourier transform of the images (bottom row). Figure 1B shows the histograms of the three images. Stimuli subtended 1.62 degrees when viewed through the AO system.

### 2.2 Polychromatic adaptive optics visual simulator

On-bench measurements and visual testing were performed with a custom-developed polychromatic AO system at the Institute of Optics (Spanish National Research Council, IO-CSIC, Madrid, Spain). The system has been described in detail in previous publications (39, 49, 50). The current configuration of the system consists of 9 different channels, of which the following were used in this study. (1) The illumination channel, with light coming from the supercontinuum laser source (SC400 femtopower 1,060 supercontinuum laser; Fianium Ltd., United Kingdom) in combination with a dual acousto-optic tunable filter module (Gooch & Housego, United Kingdom) that delivers light in multiple wavelengths through 2 different fiber outputs (visible and near-infrared). (2) The AO channel, consisting of the Hartmann-Shack wavefront sensor (microlens array 40 × 32, 3.6 mm effective diameter, centered at 1,062 nm; HASO 32 OEM, Imagine Eyes, France) and the electromagnetic deformable mirror (52 actuators, 15-mm effective diameter, 50 μm stroke; MIRAO, Imagine Eyes, France) to measure and correct the high-order aberrations (HOAs). In this study, it was used to compensate for the system aberrations and to measure and correct the aberrations of the subjects. The system aberrations were corrected at 827 nm. (3) The psychophysical channel uses a Digital Micro-Mirror Device (DMD, DLP® Discovery™ 4,100 0.7 XGA; Texas Instruments, United States) placed in a conjugate retinal plane to display visual stimuli subtending 1.62 angular degrees. The DMD was monochromatically illuminated with light coming from the supercontinuum laser source, and with white light coming from a white light fiber lamp (OSL2B—3,200 K; Thorlabs, Germany). (4) The pupil monitoring channel consists of an infrared camera conjugated to the pupil of the eye, in combination with an infrared LEDs ring. (5) The Badal optometer channel corrects for defocus and allows for through-focus psychophysical testing. All optoelectronic elements of the system were automatically controlled and synchronized by custom software written in Visual C++ and C# (Microsoft, United States) and MATLAB (MathWorks, United States).

### 2.3 On-bench testing

TF optical quality was evaluated on-bench in the same AO system using an artificial eye placed at the position of the subject’s eye using a 3-D micrometer stage. Single-pass TF retinal images of the three stimuli were collected on an artificial eye equipped with an objective lens (50.8 mm of focal length) and a CCD camera (DCC1545M, High Resolution USB2.0 CMOS Camera, Thorlabs, Germany) acting as a “retina,” in place of the subject’s eye. The stimuli were displayed in the DMD, illuminated with light from the supercontinuum laser source for monochromatic illumination and the white light lamp for polychromatic illumination, for a pupil diameter of 5 mm. Each stimulus was displayed in the DMD on a black background and projected onto the retina of the system (i.e., the CCD camera). The vergence of the system was varied from −0.75 to +0.75 D in steps of 0.25 D by changing the position of the Badal system. TF images were acquired for the wavelengths 480, 550, 633 nm and white light and for all stimuli, while correcting for the higher order aberrations of the system (RMS < 0.05 microns over the entire range).

### 2.4 *In vivo* experimental testing

To find the subjective best focus, subjects adjusted the position of the Badal system using a keyboard until the stimulus was perceived focused using the methods of limits (precision 0.01 D steps) while viewing the stimulus illuminated at a series of wavelengths in visible light (450, 480, 500, 550, 633 and 670 nm) as well as in white light. The luminance of the stimulus was approximately 20 cd/m^2^ throughout the tested spectral range (450–670 nm), therefore in the photopic range at all wavelengths. Equiluminance across wavelengths and white light was ensured during calibration. Subjects were instructed to use the keyboard to find the position where the stimulus appeared sharp. Subjects first performed a trial run using the reference wavelength of 550 nm to familiarize themselves with the test. The starting point of the Badal was randomly chosen between +1.00 and +1.50 D (placed beyond the optical infinity) beyond the subject’s best focus reported in the first trial to avoid any accommodative response (e.g., if the best focus at 550 nm in the first trial was −2.00 D, the starting point for finding the best focus at 550 nm was between −0.50 and −1.00 D). The best focus search was repeated 3 times for each wavelength. Measurements were later performed for each stimulus randomly for all wavelengths and white light. Later, subjects performed the same task but with AO correction. The state of the deformable mirror, which compensated for the ocular aberrations of each eye, was determined in a closed-loop operation at 880 nm and applied to measurements at all wavelengths (39). Aberrations were monitored throughout the experiment to ensure a residual RMS < 0.1 microns in all cases and all subjects.

The subjective best focus was obtained for each of the monochromatic and polychromatic light sources, three stimuli, in the presence of natural aberrations and with AO correction. Subjects were stabilized with a dental impression and the pupil of the eye was aligned with the optical axis of the instrument using the line of sight as a reference, while the natural pupil was viewed on the monitor with a pupil camera. An artificial pupil was used to maintain the same pupil diameter (5 mm) across subjects (subjects’ pupil size was greater than 5 mm − 5.49 ± 0.33 mm). The room lights were turned off during the measurements. Breaks were taken every 30 min, and subjects could stop if they needed additional rest. The entire experimental session lasted approximately 2 h.

### 2.5 Subjects

**Figure 2:**
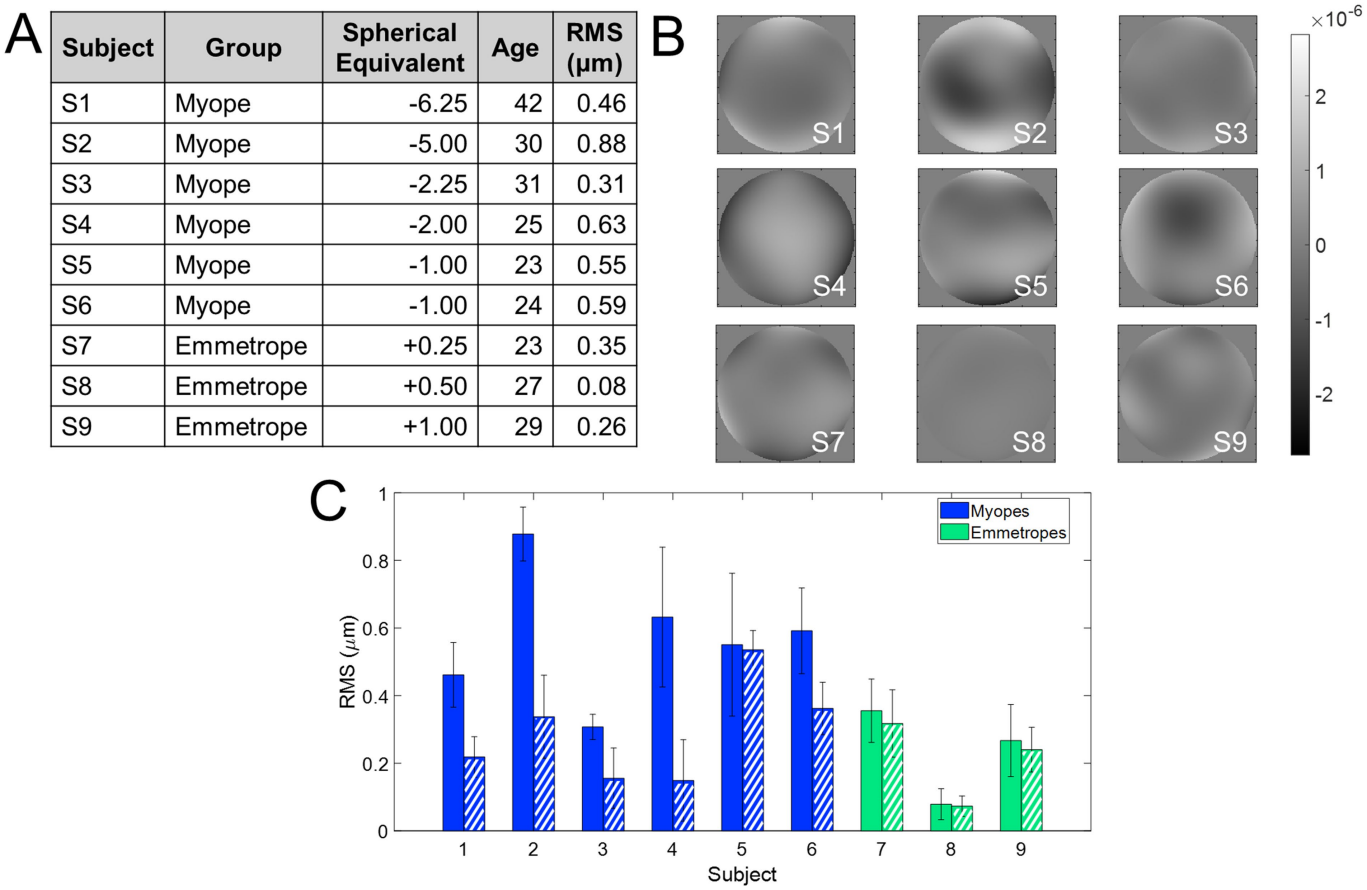
Refractive profile of the subjects enrolled in the study. **(A)** Demographics of the subjects enrolled in the study. The RMS wavefront error is with astigmatism and high-order aberrations. **(B)** Wavefront aberration maps. The scale bar is microns. **(C)** RMS wavefront error. Solid bars show the RMS with all aberrations except for piston, tilts, and defocus, and dashed bars show only high-order aberrations. Blue indicates myopes and green emmetropes.

Nine subjects were tested monocularly in the AO system. Subjects were healthy young adults (28.2 ± 6.0 years), with refractive errors ranging from −6.25 to +1.00 D (astigmatism < 0.50 D in all cases). Subjects were classified into myopes (spherical refractive error higher than −0.5 D) and emmetropes (spherical refractive error lower −0.50 and +1.00 D). Refractive error was determined from their current prescription and adjusted, if necessary, using traditional subjective refraction, i.e., the fogging technique. There was no difference in the refractive error from the current prescription in any of the subjects. The RMS of 3rd order and higher aberrations (at 5 mm pupil diameter) in the subjects ranged from 0.07 to 0.54 μm. Figure 2A summarizes the refractive and aberration profile of the subjects, ranked according to the magnitude of their refractive error.

All subjects were informed of the nature and possible consequences of the study and provided written informed consent. All protocols met the tenets of the Declaration of Helsinki and were previously approved by the Bioethics Committee of the Spanish National Research Council (CSIC).

### 2.6 Data analysis

*On-bench testing*: TF optical quality was obtained from the images of each stimulus on-bench. The image quality metric was obtained from the correlation coefficient (correlation of each of the TF images with the image in the best focus for each wavelength under the same conditions: laser power, pupil diameter, and exposure time) of the image series. Only the region of interest (where the stimulus was located in the camera image) was analyzed and converted to grayscale before estimating the correlation. The correlation metric is expected to peak at 0 D in all conditions. To provide a quantitative estimate of the degradation for both wavelength and defocus sign –myopic (negative) and hyperopic (positive)–, the absolute value of the slope of the linear regression adjustment between the negative defocus values and the correlation metric and the positive defocus values and the correlation metric was calculated. This analysis was performed considering only defocus values within the range of ±0.5 D (from −0.5 to 0.0 D for negative defocus, and from 0.0 to +0.5 D for positive defocus). The slope is an indicator of image degradation as defocus increases. A higher slope value means that the decrease in the correlation metric is greater and therefore the degradation is also greater.

*In vivo testing*: The subjective best focus in each condition was obtained directly from the Badal system readings. The average and the standard deviation across the 3 repeated measurements provided the subjective best focus and its repeatability for all conditions, respectively. In addition, the chromatic difference of focus curves was obtained from them by shifting the best focus curves on the vertical axis so that they crossed zero at 550 nm (the reference wavelength). The chromatic difference in focus (CDF) was fitted to a quadratic regression. The Green-Blue (G-Blue) difference was estimated as the chromatic difference in focus between green and blue (550 and 480 nm) using the adjustments for each refractive error group. Similarly, the Green-Red G-Red difference was estimated as the chromatic difference in focus between green and red (550 and 700 nm) using the adjustments for each refractive error group. Finally, the longitudinal chromatic aberration (LCA) was estimated by fitting these chromatic differences of focus curves within the visible region (from 470 to 700 nm). A Mann–Whitney U-test two-tailed was used to analyze the statistical significance of the differences between the results of the different conditions, and of different refractive error groups. A linear mixed model with Bonferroni correction was applied to account for multiple comparisons. The significance level was set at 5% (*p* = 0.05). MATLAB (Math-works Inc., Natick, United States) was used for analysis. Spearman correlation was used to compare psychophysical data to explore trends in visual perception.

## 3 Results

### 3.1 Through-focus on-bench image analysis

**Figure 3:**
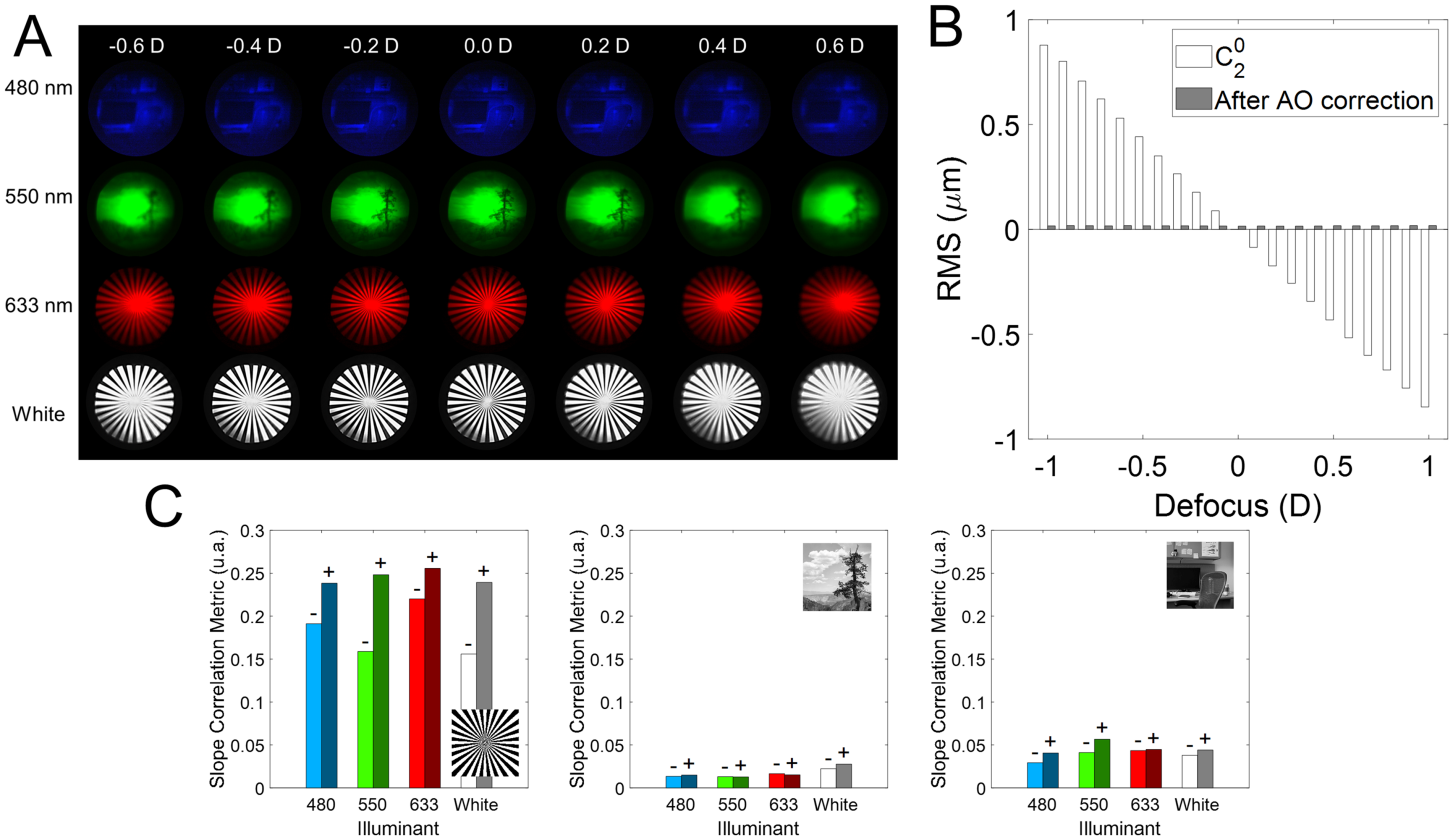
On-bench TF images analysis. **(A)** Illustrative examples of the TF images for different illumination (480, 550, 633 nm, and white light) and stimulus conditions. **(B)** Optical quality throughout the experiments. The defocus coefficient term of the wavefront at the pupil plane of the system measured with the wavefront sensor (white bars, $c_{2}^{0}$ ), and the residual RSM after AO correction (gray bars) of the HOAs + astigmatism of the system. **(C)** Absolute value of the slope of the linear regression fit of the correlation and defocus in the range ±0.50 D. A higher value of the slope indicates a greater degradation as a function of defocus. A minus sign is placed over the negative defocus slope bars (values < 0.00 D) and a positive sign over the positive defocus slope bars (values > 0.00 D).

Figure 3 shows the on-bench through-focus (TF) image series acquired for the different conditions tested in a range of 1.20 D (± 0.60 D, Figure 3A), while AO-correcting the HOAs of the system. The residual Root Mean Square (RMS) after AO-correction was less than 0.05 microns for all conditions, and only the defocus term changed when the Badal system was varied (Figure 3B). Figure 3C shows the slope of the correlation metric as a function of defocus for negative and positive defocus and all wavelengths and stimuli as a bar graph (see Data Analysis section in Methods). A higher value of the slope indicates a greater decrease in the correlation metric and therefore a greater degradation. For each wavelength, the slope for negative defocus (lighter color) is on the left, and the slope for positive defocus (darker color) is on the right. For all stimuli and wavelengths, the slope for positive defocus is higher than for myopic defocus (0.103 vs. 0.079 on average), meaning that positive defocus degrades the images slightly more than negative defocus. In addition, the slope is lower for the outdoor natural stimulus than for the other two (0.017 for outdoor vs. 0.042 for indoor and 0.213 for sunburst), indicating less degradation of the outdoor images than the binary/indoor images. The sunburst had the steepest slope and therefore the highest degradation (0.256 for positive defocus in the red wavelength).

### 3.2 Subjects’ profile

Figure 2 shows the refractive and aberration profiles of the subjects who participated in the experiment, grouped according to their refractive error (from higher myopia to higher hyperopia). Figure 2A shows the demographic information, Figure 2B the wavefront aberration map of all subjects participating in the study, and Figure 2C a comparison of the RMS of HOAs + astigmatism (solid bars) and RMS of HOAs only (dashed bars). The RMS of HOAs + astigmatism ranged from 0.08 to 0.88 μm (mean 0.46 ± 0.24 μm), whereas the RMS of HOAs only ranged from 0.08 to 0.53 μm (0.26 ± 0.14 μm).

### 3.3 Polychromatic subjective best focus

**Figure 4:**
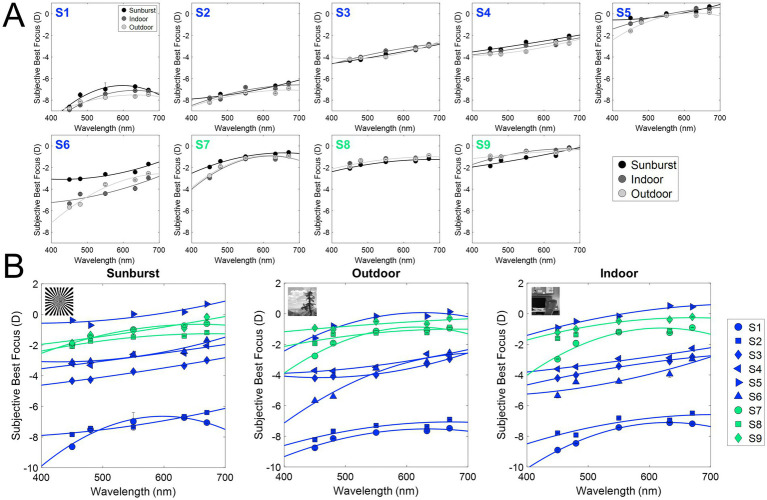
Subjective best focus for different wavelengths and stimuli in the presence of natural aberrations and after AO-correction. **(A)** Individual chromatic defocus curves (CDF) for the three stimuli tested. **(B)** Individual results for all subjects as a function of the stimulus (black, binary; dark gray, indoor gray scale; light gray, outdoor gray scale). Blue markers represent myopes (S1 to S6, ranked by increasing refractive error), and green markers represent emmetropes (S7 to S9).

Figure 4 shows the subjective best focus for all wavelengths, stimuli, and subjects measured in Experiment 1, with natural aberrations. Blue represents myopes (S1 to S6; refractive error higher than −0.50 D) and green represents emmetropes (S7 to S9; between −0.50 and 1.00 D). Figure 4A shows the subjective best focus for the three stimuli (Sunburst, black; Indoor, dark gray; Outdoor, light gray) for each wavelength, and the corresponding fit for each subject. Figure 4B shows the individual data clustered stimuli.

Similar to previous experiments using the same experimental setup (39), the variability of the best focus task was very low (0.06 D on average, both in the presence of natural aberrations, and after AO-correction). As expected, the subjective best focus for green (550 nm) was highly correlated with that for white light, both in the presence of natural aberrations (*ρ* = 0.98; slope = 1.00; y-intercept = 0.22) and after AO-correction (ρ = 0.97; slope = 0.94; y-intercept = −0.13). As expected, shorter wavelengths were focused on more negative (myopic) focus, and longer wavelengths on more positive (hyperopic) focus. Subjective best focus with natural aberrations and AO-correction was highly correlated (ρ = 0.97; slope = 0.95; y-intercept = −0.39).

**Figure 5:**
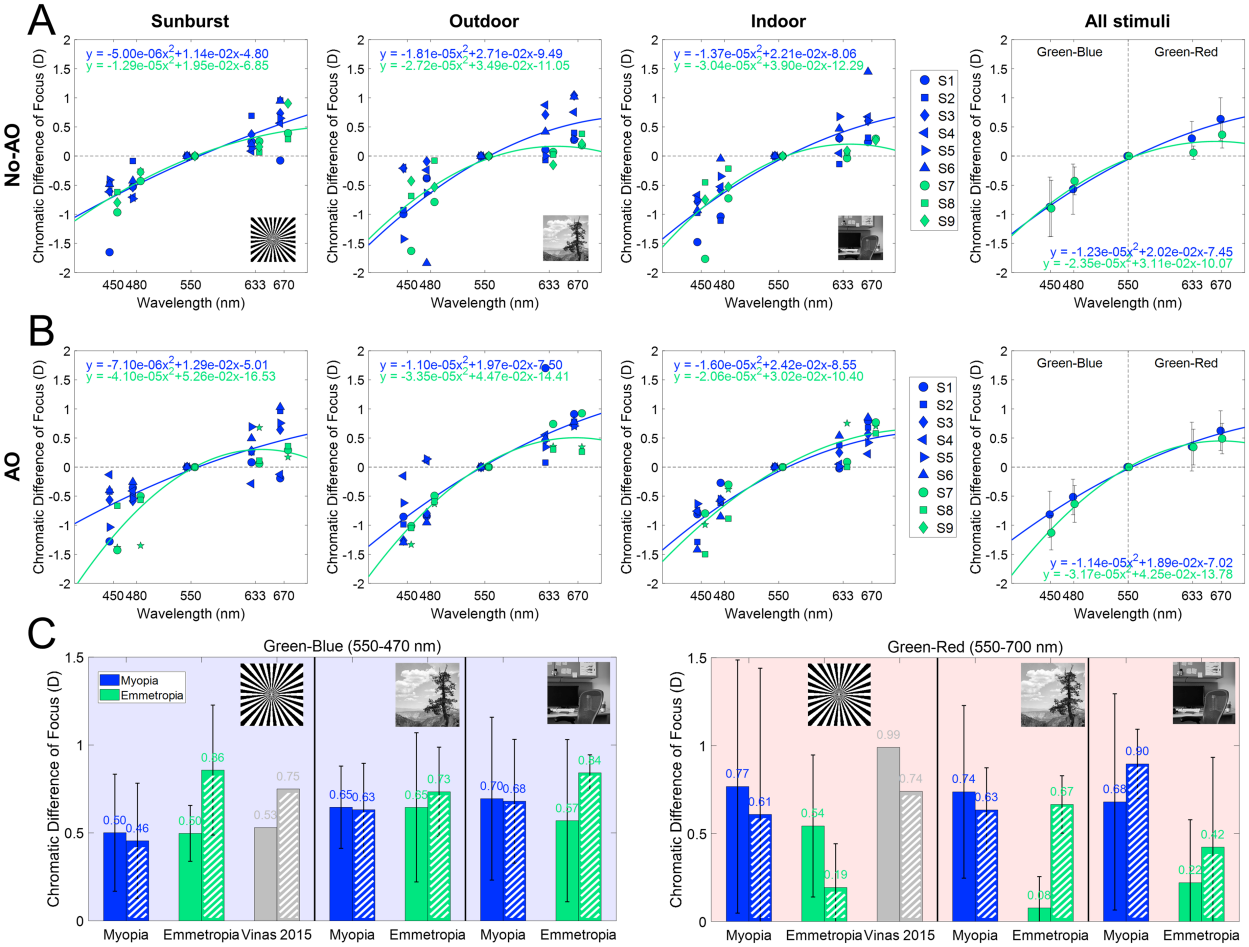
Chromatic difference of focus curves (470–700 nm). **(A)** Chromatic difference of focus curves (centered at 550 nm) for each stimulus and group. **(B)** AO-corrected chromatic difference of focus fitting curves (centered at 550 nm) for each stimulus and group. **(C)** Chromatic difference of focus for green-blue (550–470 nm) and green-red (550–700 nm) for all conditions, both with natural aberrations and with AO-correction. Dashed bars are AO correction data. Data from Vinas et al. (39) are shown as gray bars.

Figure 5 and Table 1 summarize the chromatic difference of focus (CDF) for the two refractive groups (myopes in blue; emmetropes in green) and the effect of AO-correction of natural aberrations. Figure 4A reports the subjective best focus (from Badal direct readings) obtained with the eye’s natural aberrations, and Figure 4B with AO-correction of high order aberrations. CDFs curves for emmetropes (green) remained relatively flat in the red region and becomes progressively steeper toward the blue region, whereas myopes (blue) exhibited markedly steep slopes for both the blue and red regions. A linear mixed model confirmed main effect of refractive error on CDF. In the presence of natural aberrations, CDF-Green-Blue is significantly lower than the Green-Red, whereas for myopes both are similar. AO-correction of ocular aberrations changes that partially. CDF-Green-Blue for emmetropes significantly increased, reaching higher values than myopes for the same range, whereas the increment for the Green-Red regions was lower. Myopes remained insensitive to the AO-correction effect.

**Table 1:** Summary of the chromatic difference of focus for all conditions and groups of subjects.

Condition	Spectral region	Group	Mean ± SD (D)	*p*	U	Effect size r
Natural Aberrations	Green-Blue (480–550 nm)	Emmetropes	0.22 ± 0.38	0.77	87	0.011
		Myopes	0.61 ± 0.34			
	G-Red (550–700 nm)	Emmetropes	0.58 ± 0.32	0.03^*^	124	0.08
		Myopes	0.73 ± 0.58			
AO Correction	Green-Blue (480–550 nm)	Emmetropes	0.81 ± 0.24	0.11	49	−0.06
		Myopes	0.59 ± 0.31			
	G-Red (550–700 nm)	Emmetropes	0.42 ± 0.36	0.05	119	0.071
		Myopes	0.71 ± 0.50			

Descriptive and inferential results from Mann-Whitney U tests. An * represents that the values are statistically different.

The effect of the different stimuli is shown in Figure 5. With natural aberrations (Figure 5A), myopes showed mean G-Blue and G-Red values of 0.61 ± 0.34 and 0.73 ± 0.58 D, respectively (*p* = 0.31, U = 130, effect size r = 0.061, Mann–Whitney U test) whereas emmetropes showed 0.58 ± 0.32 and 0.22 ± 0.38 D, respectively (*p* = 0.049, U = 63, effect size r = 0.108, Mann–Whitney U test), significantly higher for G-Blue than for G-Red. The inter-group difference was significant for G-Red (*p* = 0.028, U = 124, effect size r = 0.08), but not for G-Blue (*p* = 0.77, U = 87, effect size r = 0.011). These differences were greater when using natural images (0.66 D outdoor and 0.46 D indoor) than the binary stimulus (0.23 D). On average, the CDF curves at 670 nm differed by 0.51 D.

AO correction (Figure 5B) accentuated inter-group differences in the G-Blue region (−0.22 at 480 nm on average, and particularly −0.80 D for the binary stimulus) while reducing separation at G-Red region (~0D at 670 nm). Within emmetropes, G-Blue remained higher than G-Red (p = 0.04, U = 64, effect size r = 0.113, Mann–Whitney U test); within groups, G-Red still differed although not statistically (*p* = 0.05, U = 119, r = 0.071) but G-Blue did not (*p* = 0.11, U = 49, r = −0.06). In addition, no significant differences arose natural and AO conditions inside either refractive group (all *p* > 0.05 in, U ranged from 21 to 173, Mann Whitney U test). For reference, the results of a previous study (39), performed in the same system and experimental conditions are shown in the figure (gray bars): 0.53 D and 0.99 D for the G-Blue and G-Red, respectively in the presence of aberrations, and 0.75 D and 0.74 after AO-correction of HOAs. The sample in that study consisted of five young subjects (28.6 ± 1.9 years) with spherical errors ranging from 0 and −4.50 D (−1.15 ± 0.95 D).

### 3.4 Subjective longitudinal chromatic aberration

**Figure 6:**
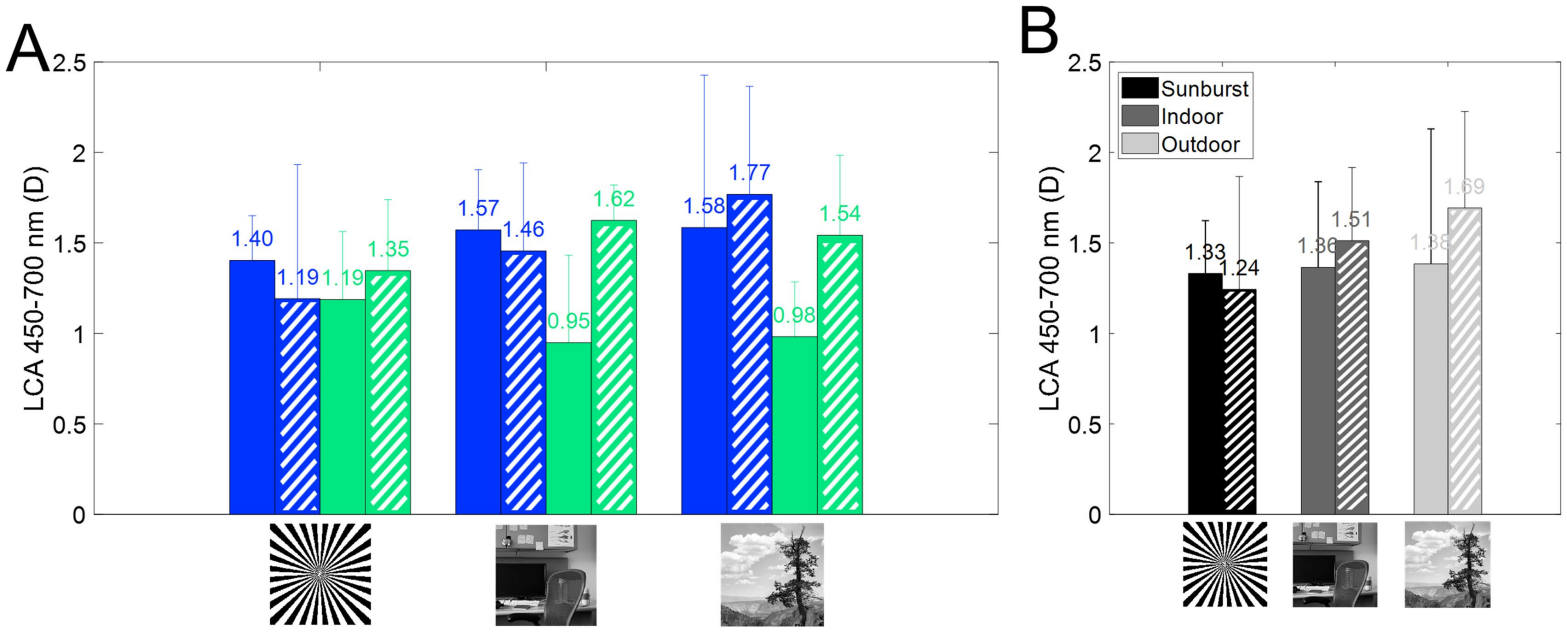
Longitudinal chromatic aberration (LCA) from 450 to 700 nm. Dashed bars represent AO correction. **(A)** Subjective LCA for myopes (blue) and emmetropes (green), for all three stimuli, with and without correction of HOAs. **(B)** Average subjective LCA as a function of the stimulus.

Subjective longitudinal chromatic aberration (LCA) was estimated from the polynomial fitting of the CDF curves for the extremes of the spectral range (from 480 to 700 nm). Figure 6A shows the average across stimuli for each refractive error group and with natural aberrations and aberrations compensated. On average, subjective LCA is similar for both refractive groups when using the binary stimuli, and AO-correction of HOAs does not modify that trend. Results are slightly different with natural images, where AO-correction of HOAs increases subjective LCA total amount, particularly for emmetropic subjects, as shown in Figure 2A. There was no statistical difference as a function of the stimulus, refractive error, or aberration condition (*p* > 0.05 for all comparisons, U ranged from 23 to 35, Mann Whitney U test). These findings were further supported by the linear mixed model, which did not identify any significant fixed effects for these factors.

## 4 Discussion

In this study, we investigated the differences in the perception of polychromatic optical cues and their dependence on refractive error, in the presence of natural aberrations and after their aberration correction, (a) to shed light on the perception of polychromatic large-and small-scale structures; (b) to test differences between emmetropic and myopic eyes in chromatic aberration and the impact of monochromatic aberrations, and (c) to investigate the visual perception of these features.

In this study, we present differences in the perception of chromatic defocus when using binary or natural stimuli, in monochromatic and polychromatic conditions. On average, G-Blue CDF (480–550 nm) was higher than G-Red CDF (555–700 nm) in emmetropes, while myopes showed the opposite trend (Figure 5). In addition, the G-Red CDF was significantly different for myopes and emmetropes (see Figure 5C). The use of different stimuli increased these differences, with natural images showing the largest differences (binary 0.23 D; indoor 0.46 D; outdoor 0.66 D). AO correction of HOAs had no effect on the G-Blue CDF on myopes, regardless of the stimuli, but increased G-Blue CDF in emmetropes when viewing the binary or the indoor stimuli. Removal of aberrations reduced the G-Red CDF obtained with the binary (emmetropes and myopes) and outdoor (myopes) stimuli, and increased it with the indoor (emmetropes and myopes) and outdoor (emmetropes) stimuli, indicating differences in perception related to the refractive profile.

### 4.1 Polychromatic subjective best focus

Overall, our results are consistent with previous reports of subjective best focus measurements at different wavelengths, when using a high-contrast stimulus, similar to the binary image used here (10, 39, 51–53), with very low variability in the subjective best focus setting (<0.10 D), both in the presence of natural aberrations and after AO-correction. Similarly, the subjective best focus task showed very low variability across subjects, stimuli, and illumination conditions, and the subjective best focus for green illuminant was correlated with that for white light, both in the presence/absence of HOAs.

The chromatic difference of focus (CDF) curve for emmetropes is flatter in the red but becomes increasingly steep in the blue, in agreement with previous studies (43). Here, myopes show steeper curves for both the blue and red regions (Figure 5). In the presence of natural aberrations, the CDF curves of emmetropes and myopes showed differences in the G-Red part of the curves, both on average and for each stimulus. These differences were greater for the CDF curves obtained with natural images than for the binary stimulus, suggesting perceptual differences associated to the refractive error and the visual information presented. The model proposed by Schaeffel and Swiatczak (54), in which the retina uses a closed-loop negative feedback system based on image defocus to modulate eye growth (16, 54), receiving contributions from spatial frequency information (stimulatory pathway) and defocus in different chromatic planes (inhibitory pathway), motivated the study of chromatic blur perception to more complex stimuli, with different refractive errors, and AO natural aberrations correction. In particular, the fact that the spatial frequency component of the visual target has been reported as a potential cause of myopia development (55) motivated the use of indoor and outdoor grayscale images.

### 4.2 Chromatic difference of focus for blue and red

When compared with the results of a previous study performed with the same system and experimental conditions (sunburst stimuli), we find similar trends in the CDF curves, but slightly higher differences within spectral ranges (0.53 D and 0.99 D for the G-Blue and G-Red, and 0.75 D and 0.74 after AO-correction of HOAs). These differences can be attributed to the sample used (young subjects, with spherical errors ranging between 0 and −4.50 D), whereas our sample is clustered by refractive error. In addition, the previous study used a linear fit, which may have misrepresented the extremes of the spectral range, especially the red region. In emmetropes, the average G-Blue CDF across stimuli was significantly higher than the average G-Red CDF (0.58 ± 0.32 > 0.22 ± 0.38 D; *p* < 0.05, U = 63, Mann–Whitney U test), whereas in myopes there was a reversed non-significant trend (0.61 ± 0.34 < 0.73 ± 0.58 D, respectively). Similarly, there were statistical differences between refractive error groups in the G-Red (*p* < 0.05, U = 124, Mann–Whitney U test), but not in the G-Blue (*p* > 0.05, Mann–Whitney U test). The chromatic difference of focus for all stimuli shows that myopes perceive red defocus differently than emmetropes. In fact, myopes do not show differences in the chromatic difference of focus between G-Blue and G-Red, whereas emmetropes do. These results suggest that the mechanism that uses chromatic defocus as an optical cue is somehow disrupted in myopes, as suggested by other authors (54).

According to the Indiana chromatic eye model (56), the G-Blue focus difference in an emmetropic eye should be larger than the G-Red (0.69 vs. 0.45 D, respectively). However, experimental data do not agree with this prediction. In a previous work (39), we showed an opposite trend (G-Blue 0.53 D and G-Red 0.99 D), and AO-correcting aberrations balance these results (0.75 D and 0.74 D, respectively). In the current study (Figure 5), the G-Blue CDF is significantly different for emmetropes and myopes when testing natural outdoor images, but not when testing indoor or binary images. Correction of natural aberrations has a significant effect on emmetropes for both binary and indoor natural images, with a significant increase in the G-Blue CDF. On the other hand, G-Red CDF was significantly lower for emmetropes than for myopes, regardless of stimulus type, with similar results for outdoor natural and binary images, and higher for indoor natural images. AO-correction of aberrations significantly increased the G-Red CDF for myopes obtained with the outdoor natural images, exceeding that of the indoor natural image. These results indicate that myopic eyes do not use the blue and red focus information, whereas emmetropes do, regardless of the stimuli, consistent with the hypothesis that the myopic retina does not respond to chromatic blur in the sense that it does not use the red-blue focus difference as a cue for emmetropization (3, 47).

### 4.3 Impact of high-order aberrations in chromatic blur

Correction of HOAs of the subjects slightly modified those trends, highlighting differences in the blue and red regions between emmetropes and myopes, especially for the binary stimulus (Figures 5A,B). The results of our study show that natural aberrations have an impact on the perceived chromatic aberration across stimuli in emmetropes, but not in myopes. AO-correction of monochromatic aberrations increases the differences in the chromatic difference of focus for the binary stimuli, while it decreases for the natural image stimuli for emmetropic eyes. Again, myopic eyes appear to be insensitive to changes in the perceived stimuli (Figure 5B). High-order aberrations in the optics of the eye can provide an odd error cue as the point spread function changes shape with the same absolute spherical equivalent refractive error, but a different sign.

### 4.4 Subjective LCA and refractive error

Although some variability was found, there was no statistical difference as a function of the stimulus, refractive error, or aberration condition (*p* > 0.05 for all comparisons). Furthermore, there is no influence of the refractive error on the magnitude of LCA (Figure 6B), either with natural aberrations or compensated aberrations. This result is in agreement with a previous report (57) showing that LCA was not correlated with refractive error (r^2^ = 0.024). The results are also slightly different for natural images, where AO-correction of HOAs increased the total amount of subjective LCA, particularly for emmetropic subjects.

### 4.5 Limitations of the study

There are limitations to this study. First, accommodation was not paralyzed during the measurements. However, the goal of this study was to test the chromatic perception under the most natural conditions possible, given the limitations of the AO system. Therefore, we chose to maintain accommodation functionality during the measurements. However, the influence of accommodation was expected to be minimal because the subjective best focus was always found from a positive defocus. The effect of accommodation on chromatic perception remains a topic for future research, as it may provide insight into the underlying perceptual mechanisms.

Another limitation of this study was the modest sample size. Measurements in an adaptive optics system are typically long and tedious, and recruiting subjects is challenging. Nonetheless, the findings of this study provide new insights into the differences in chromatic perception between myopes and emmetropes and pave the way for the development of more efficient and rapid testing methods that can be implemented with a larger sample sizes.

Overall, the results of this study suggest that the refractive profile may influence the perception of visual information with specific chromatic properties, leading to differences in the processing of these properties. However, future work should focus on disentangling other mechanisms involved in the perception of polychromatic optical cues, such as accommodation dynamics, refractive error, and age range.

## Data Availability

The original contributions presented in the study are included in the article/supplementary material, further inquiries can be directed to the corresponding author.
